# Supplementary material for: Hyperbolic matter in electrical circuits with tunable complex phases
Source: Nat Commun. 2023 Feb 4;14:622. doi: 10.1038/s41467-023-36359-6 (PMC9899218; doi:10.1038/s41467-023-36359-6)
Supplement: Supplementary file 1 — Supplementary Information [file 41467_2023_36359_MOESM1_ESM.pdf]

Supplementary Information to: Hyperbolic Matter in Electrical Circuits with Tunable Complex Phases

Anffany Chen [1,2] Hauke Brand,[3] Tobias Helbig,[4] Tobias Hofmann,[4] Stefan Imhof,[3] Alexander Fritzsche,[5,4] Tobias Kießling,[3] Alexander Stegmaier,[4] Lavi K. Upreti,[4] Titus Neupert,[6] Tomáš Bzdušek,[7,6] Martin Greiter,[4] Ronny Thomale,[4] and Igor Boettcher [1,2,*]

[1] *Department of Physics, University of Alberta, Edmonton, Alberta T6G 2E1, Canada*
[2] *Theoretical Physics Institute, University of Alberta, Edmonton, Alberta T6G 2E1, Canada*
[3] *Physikalisches Institut, Universität Würzburg, 97074 Würzburg, Germany*
[4] *Institut für Theoretische Physik und Astrophysik, Universität Würzburg, 97074 Würzburg, Germany*
[5] *Institut für Physik, Universität Rostock, 18059 Rostock, Germany*
[6] *Department of Physics, University of Zurich, Winterthurerstrasse 190, 8057 Zurich, Switzerland*
[7] *Condensed Matter Theory Group, Paul Scherrer Institute, 5232 Villigen PSI, Switzerland*

(Dated: January 13, 2023)

CONTENTS

Supplementary Section S I Hyperbolic Bloch-Wave Hamiltonians 1

Supplementary Section S II Extensive Boundary of Hyperbolic Flakes 5

Supplementary Section S III DOS Comparisons Between HBT and Finite Lattices 6
Supplementary Discussion: HBT vs. Hyperbolic Flakes 6
Supplementary Discussion: HBT vs. Hyperbolic Regular Maps 8
Supplementary Discussion: HBT vs. Higher-dimensional Euclidean lattices 9

Supplementary Section S IV Nodal Region and Dirac Hamiltonian of Hyperbolic Graphene 11

Supplementary Section S V Bulk-Boundary correspondence 12

Supplementary Section S VI Floquet Band Gaps in Hyperbolic Graphene 12
Supplementary Discussion: Brief Review of Floquet Formalism 13
Supplementary Discussion: Time-Periodic Hyperbolic Graphene 13

Supplementary Section S VII Derivation of the circuit Laplacian of the phase element 16

Supplementary Section S VIII Experimental realization of the phase element 18

Supplementary References 21

SUPPLEMENTARY SECTION S I HYPERBOLIC BLOCH-WAVE HAMILTONIANS

In this supplementary section, we show that every eigenstate of the Bloch-wave Hamiltonian realized by the unit-cell circuit is a solution to the Schrödinger equation on the infinite lattice. Our derivation includes the description of an explicit algorithm for constructing the Bloch-wave Hamiltonian $H(\mathbf{k})$ for a given lattice that decomposes into Bravais lattice and unit cells.

Consider an infinite $\{p, q\}$ hyperbolic lattice that can be split into unit cells, each containing N sites, that are arranged in a hyperbolic Bravais lattice $\{p_B, q_B\}$ [1]. The translation symmetry group of the Bravais lattice is a Fuchsian group Γ generated by translation operators $\{T_1, T_2, ..., T_{p_B}\}$, each translating a unit cell to one of its p_B surrounding unit cells. Note that the generators are not mutually independent. They contain inverses and satisfy certain identities, reducing the number of independent generators to $2g$ where g is an integer > 1. This important feature of hyperbolic lattices is discussed at length in Refs. [1, 2].

The tight-binding Hamiltonian with nearest-neighbour hopping is

$$\mathcal{H} = -J \sum_{\langle i,j \rangle} (c_i^\dagger c_j + c_j^\dagger c_i), \tag{S1}$$

where i, j are the site indices and J is the hopping amplitude. The single-particle states are solutions to the time-independent Schrödinger equation $\mathcal{H}|\psi\rangle = E|\psi\rangle$. We expand $|\psi\rangle$ in the position basis

$$|\psi\rangle = \sum_i \psi(z_i)|i\rangle = \sum_i \psi(z_i) c_i^\dagger |0\rangle, \tag{S2}$$

where the coefficients $\psi(z_i) = \langle i|\psi\rangle$ can be understood as the wavefunction of the state $|\psi\rangle$ evaluated at coordinate z_i on the Poincaré disk. This gives one equation for each site i,

$$E\psi(z_i) = -J \sum_{m \in n(z_i)} \psi(z_m), \tag{S3}$$

where $n(z_i)$ is the set of q neighbours of site i. Without loss of generality, let us focus on the equation for an arbitrary site i. Some of its neighbours belong to its unit cell, denoted U_i, and others belong to nearby unit cells. For clarity of notation, we re-label the coordinates of the sites in U_i as $u_1^{(i)}, u_2^{(i)}, ..., u_N^{(i)}$, with $u_1^{(i)} \equiv z_i$. Due to the translation symmetry of the Bravais lattice, one can use U_i as a reference unit cell and express any neighbouring site $m \in n(u_1^{(i)})$ as $\gamma_m u_{\alpha_m}^{(i)}$ for some index $\alpha_m \in \{1, 2, ..., N\}$ and some translation operator $\gamma_m \in \Gamma$. The choice of (γ_m, α_m) is unique. If m happens to be in U_i, then γ_m is the identity element, but in general it is a product of generators. Thus Eq. (S3) for site i can be written as

$$E\psi(u_1^{(i)}) = -J \sum_{m \in n(u_1^{(i)})} \psi(\gamma_m u_{\alpha_m}^{(i)}). \tag{S4}$$

Since $\mathcal{H}$ is invariant under Γ, all of its eigenstates belong to the irreducible representations of Γ. Here we focus on the $U(1)$ representations. In other words, our eigenstates satisfy the Bloch condition [2]

$$\psi_{\mathbf{k}}(T_\mu z) = e^{ik_\mu} \psi_{\mathbf{k}}(z), \tag{S5}$$

where k_μ is the generalized crystal momentum corresponding to generator T_μ. Based on how $|\psi\rangle$ transforms under the generators, it is labelled by $\mathbf{k} = (k_1, k_2, ..., k_{2g})$, noting that only $2g$ generators are independent. Applying Eq. (S5) recurrently gives the Bloch condition for a generic translation operator $\gamma = T_{\mu_1} T_{\mu_2} ... T_{\mu_\ell}$,

$$\psi_{\mathbf{k}}(\gamma z) = e^{ik_{\mu_1}} e^{ik_{\mu_2}} ... e^{ik_{\mu_\ell}} \psi_{\mathbf{k}}(z) = e^{i(k_{\mu_1} + k_{\mu_2} + ... + k_{\mu_\ell})} \psi_{\mathbf{k}}(z). \tag{S6}$$

Equation (S6) allows us to write Eq. (S4) in terms of the wavefunction coefficients within the reference unit cell U_i:

$$E\psi(u_1^{(i)}) = -J \sum_{m \in n(u_1^{(i)})} e^{i\phi_{\mathbf{k}}(\gamma_m)} \psi(u_{\alpha_m}^{(i)}) \equiv -J \sum_{m \in n(u_1^{(i)})} A_{1,\alpha_m}(\mathbf{k}) \psi(u_{\alpha_m}^{(i)}) \tag{S7}$$

with complex phase factors dependent on γ_m as prescribed by Eq. (S6). We repeat the same procedure for the other sites $u_2^{(i)}$,...,$u_N^{(i)}$ in U_i until we obtain a total of N equations, giving rise to a $N \times N$ adjacency matrix $A(\mathbf{k})$ describing the complex-valued edges connecting sites within U_i. Due to the translation symmetry of the Bravais lattice, we would obtain an identical $A(\mathbf{k})$ (up to a change of basis) had we chosen a difference site i to begin with. Therefore the spectral problem of the tight-binding model on the infinite $\{p, q\}$ hyperbolic lattice is now reduced to diagonalizing the $\mathbf{k}$-dependent Hamiltonian $H(\mathbf{k}) = -JA(\mathbf{k})$. Note that by replacing the Fuchsian group with $\mathbb{Z} \times \mathbb{Z}$, the above derivation applies to two-dimensional Euclidean lattices and reproduces the conventional band theory obtained via Fourier transformations.

While Eq. (S7) may seem complicated, in practice it is straightforward to construct $A(\mathbf{k})$ for a given hyperbolic lattice if its Bravais lattice and unit cells are known. One starts by writing down the Poincaré-disk coordinates of all the sites in the central unit cell and constructing the PSU(1, 1) matrix representation of the Fuchsian group generators T_μ (see Ref. [1] for detailed discussion on the geometry of hyperbolic lattices and the Fuchsian group generators). Then for each unit-cell site u_n, one performs a numerical ground search for the specific product of generators that, when applied to some u_m in the unit cell, yields a site that has the right hyperbolic distance from u_n to be a nearest

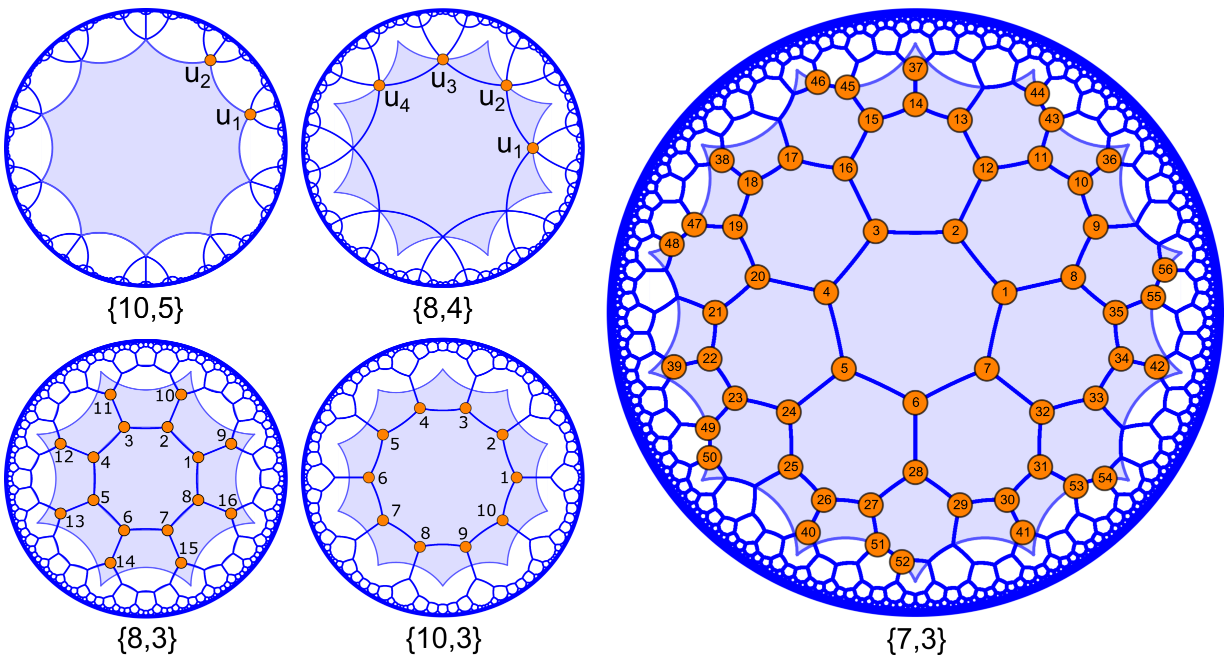

FIG. S1. **Unit cells and Bravais lattices.** The lattices used in this work can be decomposed into Bravais lattices and unit cells of various sizes. Here we represent them by the Poincaré disk model. The unit cells are indicated by the shaded regions with N sites shown in orange. The numbers correspond to the order of rows/columns in the Hamiltonians.

neighbor. (For each site u_n, q neighbors exist.) These products of generators then give rise to the complex phases according to Eq. (S6).

As an example, let us consider the $\{10, 5\}$ hyperbolic lattice, which can be decomposed into a $\{p_B, q_B\} = \{10, 5\}$ Bravais lattice with 2 sites in each unit cell, labelled u_1 and u_2 as shown in Fig. S1. Their coordinates in the Poincaré disk are $u_1 = r_0 e^{i\pi/10}$ and $u_2 = r_0 e^{i3\pi/10}$ with $r_0 = \sqrt{\cos(3\pi/10)/\cos(\pi/10)}$. The Fuchsian group generators are

$$T_1 = \frac{1}{\sqrt{(1-\sigma^2)}} \begin{pmatrix} 1 & \sigma \\ \sigma & 1 \end{pmatrix}, \ \sigma = \sqrt{\frac{\cos(2\pi/p_B) + \cos(2\pi/q_B)}{1 + \cos(2\pi/q_B)}} \tag{S8}$$

and

$$T_\mu = R(2\pi(\mu-1)/p_B) T_1 R(-2\pi(\mu-1)/p_B) \text{ for } \mu = 1, ..., p_B/2. \tag{S9}$$

Here $R(\theta) = \begin{pmatrix} e^{i\theta/2} & 0 \\ 0 & e^{-i\theta/2} \end{pmatrix}$ is the rotation matrix. Their action on the complex coordinate z is defined as

$$\begin{pmatrix} a & b \\ c & d \end{pmatrix} z := \frac{az+b}{cz+d}. \tag{S10}$$

Furthermore, they satisfy the identity

$$T_5 = -T_1^{-1} T_2 T_3^{-1} T_4, \tag{S11}$$

so there are four independent generators, corresponding to a four-component crystal momentum.

Computational search finds that the nearest neighbours of u_1 are u_2, $T_1 T_2^{-1} u_2$, $T_2 T_3^{-1} u_2$, $T_1 T_4 T_3^{-1} u_2$, and $T_2 T_3^{-1} T_4 T_3^{-1} u_2$. Running through permutations of generators acting on u_1 or u_2, we identify the neighbours by calculating their hyperbolic distances from u_1, which must equal the hyperbolic distance between u_1 and u_2 (known neighbours). Consequently $A_{1,1}(\mathbf{k}) = 0$ and $A_{1,2}(\mathbf{k}) = 1 + e^{i(k_1-k_2)} + e^{i(k_2-k_3)} + e^{i(k_1-k_3+k_4)} + e^{i(k_2-2k_3+k_4)}$. Since the adjacency matrix is Hermitian, $A_{2,1}(\mathbf{k}) = A^*_{1,2}(\mathbf{k})$ and $A_{2,2}(\mathbf{k}) = 0$. Thus the Bloch-wave Hamiltonian of $\{10, 5\}$ hyperbolic lattice is

$$H_{\{10,5\}}(\mathbf{k}) = -J \begin{pmatrix} 0 & 1 + e^{\mathrm{i}(k_1-k_2)} + e^{\mathrm{i}(k_2-k_3)} + e^{\mathrm{i}(k_1-k_3+k_4)} + e^{\mathrm{i}(k_2-2k_3+k_4)} \\ \text{c.c.} & 0 \end{pmatrix}. \tag{S12}$$

Row	Col.	Entry	Row	Col.	Entry	Row	Col.	Entry	Row	Col.	Entry	Row	Col.	Entry
1	2	-1	10	11	-1	20	21	-1	30	31	-1	43	44	-1
	7	-1		36	-1	21	22	-1		41	-1	44	50	$-e^{ik_1}$
	8	-1	11	12	-1		55	$-e^{ik_6}$	31	32	-1		52	$-e^{ik_2}$
2	3	-1		43	-1	22	23	-1		53	-1	45	46	-1
	12	-1	12	13	-1		39	-1	32	33	-1	46	52	$-e^{ik_3}$
3	4	-1	13	14	-1	23	24	-1	33	34	-1		54	$-e^{ik_4}$
	16	-1		51	$-e^{ik_2}$		49	-1		47	$-e^{-ik_5}$	47	48	-1
4	5	-1	14	15	-1	24	25	-1	34	35	-1	48	54	$-e^{ik_5}$
	20	-1		37	-1	25	26	-1		42	-1		56	$-e^{ik_6}$
5	6	-1	15	16	-1		43	$-e^{-ik_1}$	35	55	-1	49	50	-1
	24	-1		45	-1	26	27	-1	36	39	$-e^{-ik_7}$	50	56	$-e^{ik_7}$
6	7	-1	16	17	-1		40	-1		40	$-e^{ik_1}$	51	52	-1
	28	-1	17	18	-1	27	28	-1	37	40	$-e^{ik_2}$	53	54	-1
7	32	-1		53	$-e^{ik_4}$		51	-1		41	$-e^{ik_3}$	55	56	-1
8	9	-1	18	19	-1	28	29	-1	38	41	$-e^{ik_4}$			
	35	-1		38	-1	29	30	-1		42	$-e^{ik_5}$			
9	10	-1	19	20	-1		45	$-e^{-ik_3}$	39	42	$-e^{ik_6}$			
	49	$-e^{-ik_7}$		47	-1									

TABLE S1. **Bloch-wave Hamiltonian for $\{7,3\}$-lattice.** Nonzero entries in the 56×56 Bloch-wave Hamiltonian of the {7, 3} lattice, in units of hopping amplitude J. For brevity, we only list the entries in the upper-triangular part of the Hermitian matrix. The momentum space is six-dimensional; the extra momentum component k_7 is given by $k_7 = -k_1+k_2-k_3+k_4-k_5+k_6$.

The Hamiltonian takes a simpler form

$$H_{\{10,5\}}(\mathbf{K}) = -J \begin{pmatrix} 0 & 1+e^{\mathrm{i}K_1}+e^{\mathrm{i}K_2}+e^{\mathrm{i}K_3}+e^{\mathrm{i}K_4} \\ \text{c.c.} & 0 \end{pmatrix} \tag{S13}$$

in the new basis $\mathbf{K} = M\mathbf{k}$ with

$$M = \begin{pmatrix} 1 & -1 & 0 & 0 \\ 0 & 1 & -1 & 0 \\ 1 & 0 & -1 & 1 \\ 0 & 1 & -2 & 1 \end{pmatrix}, \ \det(M) = 1. \tag{S14}$$

In the following, we list the other hyperbolic Bloch-wave Hamiltonians used in this work.

$\{8,4\}$ lattice – The Bravais lattice is $\{8,8\}$. As shown in Fig. S1, the unit cell has four sites: $u_1 = r_0$, $u_2 = r_0 e^{i\pi/4}$, $u_3 = r_0 e^{i\pi/2}$, and $u_4 = r_0 e^{i3\pi/4}$ with $r_0 = \sqrt{\cos(3\pi/8)/\cos(\pi/8)}$. The hyperbolic Brillouin zone is 4-dimensional, corresponding to four independent Fuchsian group generators $T_1, ..., T_4$ as defined in Eqs. (S8) and (S9) with $p_B = q_B = 8$. The four nearest neighbours of u_1 are u_2, $T_4^{-1}u_4$, $T_1 u_4$, and $T_1 T_2^{-1} u_2$. The neighbours of u_2 are u_1, $T_2 T_1^{-1} u_1$, u_3, and $T_2 T_3^{-1} u_3$. The neighbours of u_3 are u_2, $T_3 T_2^{-1} u_2$, u_4, and $T_3 T_4^{-1} u_4$. The neighbours of u_4 are u_3, $T_1^{-1} u_1$, $T_4 u_1$, and $T_4 T_3^{-1} u_3$. Therefore the Bloch-wave Hamiltonian is

$$H_{\{8,4\}}(\mathbf{k}) = -J \begin{pmatrix} 0 & 1+e^{\mathrm{i}(k_1-k_2)} & 0 & e^{\mathrm{i}k_1}+e^{-\mathrm{i}k_4} \\ 1+e^{-\mathrm{i}(k_1-k_2)} & 0 & 1+e^{\mathrm{i}(k_2-k_3)} & 0 \\ 0 & 1+e^{-\mathrm{i}(k_2-k_3)} & 0 & 1+e^{\mathrm{i}(k_3-k_4)} \\ e^{-\mathrm{i}k_1}+e^{\mathrm{i}k_4} & 0 & 1+e^{-\mathrm{i}(k_3-k_4)} & 0 \end{pmatrix}. \tag{S15}$$

$\{7,3\}$ lattice – The Bravais lattice is $\{14,7\}$ with 56 sites in each unit cell (see Fig. S1). There are six independent Fuchsian-group generators, resulting in a six-dimensional hyperbolic Brillouin zone. The nonzero entries of the 56×56 Bloch-wave Hamiltonian are listed in Table S1.

$\{8,3\}$ lattice – The Bravais lattice is $\{8,8\}$ with unit cell size $N = 16$ (see Fig. S1). The hyperbolic Brillouin zone is

4-dimensional. The Bloch-wave Hamiltonian can be read off Fig 1f:

$$H_{\{8,3\}}(\mathbf{k}) = -J \begin{pmatrix} 0&1&0&0&0&0&0&1&1&0&0&0&0&0&0&0 \\ 1&0&1&0&0&0&0&0&0&1&0&0&0&0&0&0 \\ 0&1&0&1&0&0&0&0&0&0&1&0&0&0&0&0 \\ 0&0&1&0&1&0&0&0&0&0&0&1&0&0&0&0 \\ 0&0&0&1&0&1&0&0&0&0&0&0&1&0&0&0 \\ 0&0&0&0&1&0&1&0&0&0&0&0&0&1&0&0 \\ 0&0&0&0&0&1&0&1&0&0&0&0&0&0&1&0 \\ 1&0&0&0&0&0&1&0&0&0&0&0&0&0&0&1 \\ 1&0&0&0&0&0&0&0&0&0&0&e^{\mathrm{i}k_1}&0&e^{\mathrm{i}k_2}&0&0 \\ 0&1&0&0&0&0&0&0&0&0&0&0&e^{\mathrm{i}k_2}&0&e^{\mathrm{i}k_3}&0 \\ 0&0&1&0&0&0&0&0&0&0&0&0&0&e^{\mathrm{i}k_3}&0&e^{\mathrm{i}k_4} \\ 0&0&0&1&0&0&0&0&e^{-\mathrm{i}k_1}&0&0&0&0&0&e^{\mathrm{i}k_4}&0 \\ 0&0&0&0&1&0&0&0&0&e^{-\mathrm{i}k_2}&0&0&0&0&0&e^{-\mathrm{i}k_1} \\ 0&0&0&0&0&1&0&0&e^{-\mathrm{i}k_2}&0&e^{-\mathrm{i}k_3}&0&0&0&0&0 \\ 0&0&0&0&0&0&1&0&0&e^{-\mathrm{i}k_3}&0&e^{-\mathrm{i}k_4}&0&0&0&0 \\ 0&0&0&0&0&0&0&1&0&0&e^{-\mathrm{i}k_4}&0&e^{\mathrm{i}k_1}&0&0&0 \end{pmatrix}. \tag{S16}$$

$\{10, 3\}$ lattice – The Bravais lattice is $\{10, 5\}$ with unit cell size $N = 10$ (see Fig. S1). The hyperbolic Brillouin zone is 4-dimensional. The Bloch-wave Hamiltonian follows from Fig. 3 in Ref. [1]:

$$H_{\{10,3\}}(\mathbf{k}) = -J \begin{pmatrix} 0&1&0&0&0&e^{\mathrm{i}k_1}&0&0&0&1 \\ 1&0&1&0&0&0&e^{\mathrm{i}k_2}&0&0&0 \\ 0&1&0&1&0&0&0&e^{\mathrm{i}k_3}&0&0 \\ 0&0&1&0&1&0&0&0&e^{\mathrm{i}k_4}&0 \\ 0&0&0&1&0&1&0&0&0&e^{\mathrm{i}k_5} \\ e^{-\mathrm{i}k_1}&0&0&0&1&0&1&0&0&0 \\ 0&e^{-\mathrm{i}k_2}&0&0&0&1&0&1&0&0 \\ 0&0&e^{-\mathrm{i}k_3}&0&0&0&1&0&1&0 \\ 0&0&0&e^{-\mathrm{i}k_4}&0&0&0&1&0&1 \\ 1&0&0&0&e^{-\mathrm{i}k_5}&0&0&0&1&0 \end{pmatrix} \tag{S17}$$

where $k_5 = -k_1 + k_2 - k_3 + k_4$.

SUPPLEMENTARY SECTION S II EXTENSIVE BOUNDARY OF HYPERBOLIC FLAKES

The boundary of a hyperbolic lattice with open boundary condition is not negligible even in the limit of large system size (unlike Euclidean lattices). This can be understood in the continuum limit. A hyperbolic circle of radius r on the Poincaré disk has circumference $C_H = 2\pi R \sinh(r/R)$ and area $A_H = 4\pi R^2 \sinh^2(r/2R)$, where $R = 1/\sqrt{-K}$ and K is the Gaussian curvature. The ratio $C_H/A_H = \coth(r/2R)/R$ approaches $1/R$ as $r \to \infty$. Table S2 lists the number of total/boundary sites for the lattices we considered. The boundary sites consist of those with coordination number less than q. The boundary-to-total ratio indeed approaches a constant as system size increases. Furthermore, this ratio is higher for lattices with higher curvature per plaquette, which we derive below.

Each p-sided polygon in a $\{p, q\}$ lattice can be divided into $2p$ right triangles of the same size by lines passing through the center of the polygon. The angles in each right triangle are $\pi/2$, π/p, and π/q. The area of a hyperbolic triangle is given by

$$A_\triangle = (\pi - \theta_\triangle)R^2 \tag{S18}$$

where $\theta_\triangle$ is the total internal angle and R is the curvature radius. Here $\theta_\triangle = \pi/2 + \pi/p + \pi/q$. The total area of the polygon is then

$$A_{\text{poly}} = 2pA_\triangle = -2p\Big(\frac{\pi}{2} - \frac{\pi}{p} - \frac{\pi}{q}\Big)/K \tag{S19}$$

where we have used $R = 1/\sqrt{-K}$. Rearranging the equation gives the curvature per plaquette/polygon:

$$\kappa \equiv KA_{\text{poly}} = -p\pi\Big(1 - \frac{2}{p} - \frac{2}{q}\Big). \tag{S20}$$

	κ		2-shell	3-shell	4-shell	5-shell
$\{7,3\}$	$-\pi/3$	total	35	112	315	847
		edge	21	56	147	385
		ratio	0.60	0.50	0.47	0.45
$\{8,3\}$	$-2\pi/3$	total	48	200	768	2888
		edge	32	120	448	1672
		ratio	0.67	0.60	0.58	0.58
$\{10,3\}$	$-4\pi/3$	total	80	490	2880	–
		edge	60	350	2040	–
		ratio	0.75	0.71	0.71	–
$\{8,4\}$	-2π	total	56	336	1968	–
		edge	48	280	1632	–
		ratio	0.86	0.83	0.83	–
$\{10,5\}$	-4π	total	90	800	7040	–
		edge	90	790	6940	–
		ratio	1.00	0.99	0.99	–

TABLE S2. **Bulk-to-edge-site ratio.** The ratio between boundary and total sites in an open hyperbolic lattice approaches a nonzero constant as system size increases. Here the boundary sites are defined as vertices with less than q neighbors. Lattices with larger curvature per plaquette κ have particularly significant boundary region.

SUPPLEMENTARY SECTION S III DOS COMPARISONS BETWEEN HBT AND FINITE LATTICES

The Bloch-wave Hamiltonians are constructed under the assumption that the energy eigenstates of hyperbolic lattices behave like Bloch waves, such that they acquire a U(1) phase factor from one unit cell to the other. Due to the non-Abelian nature of the Fuchsian translation group Γ, eigenstates which transform as higher-dimensional representations of Γ can also be present. Exactly how much of the full energy spectrum is captured by the Bloch eigenstates is an ongoing research problem [3]. One obvious approach to test the validity of the Bloch-wave assumption is to compare the energy spectra obtained by exact diagonalization of real-space, finite-sized hyperbolic lattices with those obtained from the Bloch-wave Hamiltonians. The main challenge is to eliminate the significant boundary effect. For a finite two-dimensional Euclidean lattice, the boundary becomes negligible at large system size. On the other hand, the boundary ratio of a hyperbolic lattice remains significant regardless of the system size (see Supplementary Sec. S II and Table S2).

In this supplementary section, we report two methods for isolating the bulk physics of finite hyperbolic lattices. We show that the resulting bulk density-of-states (bulk-DOS) is generally in good agreement with the DOS computed from the Bloch-wave Hamiltonians. We also discuss possible causes for the discrepancies.

Supplementary Discussion: HBT vs. Hyperbolic Flakes

We consider the following $\{p,\, q\}$ hyperbolic lattices: $\{7,\, 3\}$, $\{8,\, 3\}$, $\{10,\, 3\}$, $\{8,\, 4\}$ and $\{10,\, 5\}$. We use the shell-construction method [1, 4] to numerically tessellate p-gons on the Poincaré disk. Starting with a central polygon, we attach polygons to all the outer edges and repeat this procedure until the desired system size is reached. Any redundant vertices are then removed. The adjacency matrix $\mathcal{A}$ of the resulting graph is used to define the nearest-neighbour hopping terms. Numerical diagonalization of -$\mathcal{A}$ yields a discrete energy spectrum E_n in units of the hopping amplitude J and eigenvectors $\psi_n(z_i)$, where z_i are the Poincaré coordinates of the lattice sites.

To meaningfully compare with the Bloch-wave Hamiltonians, we want to discard states localized at the boundary. However, a closer look at the spatial distribution of the eigenstates reveals that while some can be categorized as either bulk or boundary states, others have significant probability density in both regions. Instead, we define the bulk bulk-DOS

$$\rho_{\text{bulk}}(\epsilon) = \sum_{z\in\Lambda_{\text{bulk}}} \left(\sum_{n\in\mathcal{N}_\epsilon} |\psi_n(z)|^2 \right) \tag{S21}$$

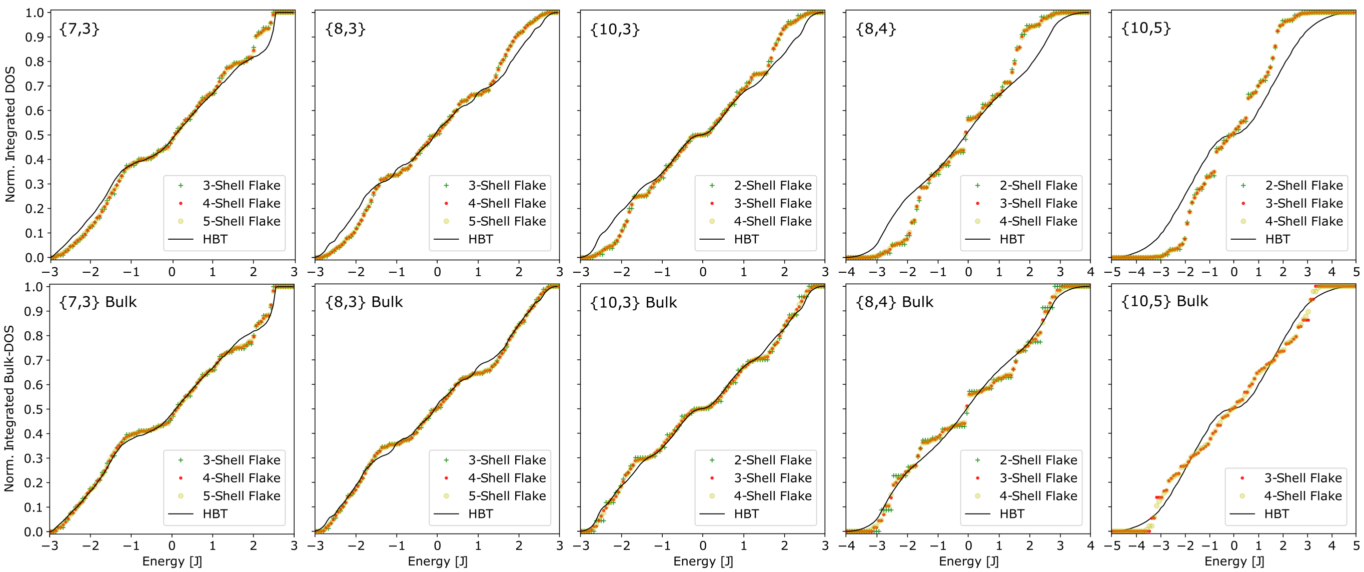

FIG. S2. **HBT vs. Hyperbolic Flakes**. The top panels compare the normalized integrated DOS obtained from Bloch-wave Hamiltonians and finite lattices in flake geometry. In the bottom panels, the boundary effect of the latter is effectively removed by using the bulk-DOS defined in Eq. (S21). For all lattices in consideration, both total DOS and bulk-DOS are independent of the system size, indicated by the number of shells used in the lattice construction. (Note that the 2-shell {10, 5} lattice has no bulk sites, so its bulk-DOS is undefined.) The efficacy of the boundary removal is most apparent in the low-energy region, where the linear growth in the integrated DOS is restored as dictated by Weyl's law [7]. For lattices {7, 3}, {8, 3}, and {10, 3}, the bulk-DOS of finite lattices agrees very well with the band-theoretical prediction. On the other hand, lattices {8, 4} and {10, 5} demonstrate a stronger discrepancy.

to effectively remove the boundary contribution to the total DOS of a hyperbolic flake. Here Λ_{bulk} is the set of lattice sites in the bulk region and $\mathcal{N}_\epsilon$ is the set of eigenstates with energies between ϵ and $\epsilon+\delta\epsilon$. We define the bulk region as all the sites with coordination number equal to q, i.e. having a complete set of q nearest neighbors. Similar quantities involving summation of local DOS over the boundary region have been used to detect topological boundary states in aperiodic systems [5, 6].

Equation (S21) is a useful quantity for isolating bulk physics for the following reasons. First of all, we find that the normalized $\rho_{\text{bulk}}(\epsilon)$ is independent of system size (see Supplementary Fig. S2). Secondly, the low-energy/long-wavelength region of $\rho_{\text{bulk}}(\epsilon)$ agrees with the continuum limit of infinite lattices. According to Weyl's law, which governs the spectral properties of the hyperbolic Laplacian in the continuum, the DOS on a two-dimensional surface without boundary is constant to leading order[7]. We observe that $\rho_{\text{bulk}}(\epsilon)$ is nearly constant in the low-energy region, successfully removing the edge contribution from the total DOS. Note that we always plot the normalized integrated DOS

$$P_{\text{bulk}}(E) = \frac{\int_{-q}^{E} \rho_{\text{bulk}}(\epsilon)\; d\epsilon}{\int_{-q}^{q} \rho_{\text{bulk}}(\epsilon)\; d\epsilon} \tag{S22}$$

because it smoothens numerical fluctuations in the DOS histograms. Constant $\rho_{\text{bulk}}(\epsilon)$ translates to linear growth in $P_{\text{bulk}}(E)$.

For each lattice in consideration, the Bloch-wave Hamiltonian is constructed following the procedure detailed in Supplementary Section "Hyperbolic Bloch-Wave Hamiltonians", where we also explicitly define all the Hamiltonians used in this work. The energy spectrum is a compilation of the eigenvalues of the Bloch-wave Hamiltonian on a fine grid of **k**-points in the Brillouin zone. It is then used to compute the normalized DOS for comparison with finite lattices.

As shown in Supplementary Fig. S2, the agreement between $\rho_{\text{bulk}}(\epsilon)$ and DOS obtained from the Bloch-wave Hamiltonian is excellent for lattices {7, 3}, {8, 3}, and {10, 3}. The agreement is not as good for {8, 4} and {10, 5}, but is nevertheless a significant improvement over the comparison without boundary effect removed. The differences in the comparison are generally caused by a combination of (i) omission of eigenstates in higher-dimensional representations of Γ and (ii) contribution to $\rho_{\text{bulk}}(\epsilon)$ by edge states penetrating deep into the bulk. The larger discrepancy for {8, 4} and {10, 5} lattices may be due to the high boundary ratios of their flakes (see Table S2), rendering them unsuitable

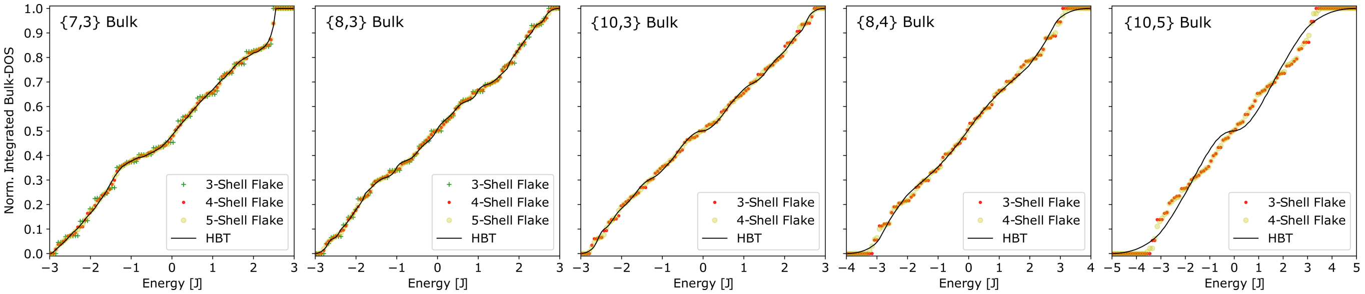

FIG. S3. **Alternative definition of bulk.** Keeping a smaller bulk region by defining the outermost two shells as the boundary improves the DOS comparison for lattices {7, 3} and {8, 3} significantly. However for lattices with higher curvature, corresponding to rapid inflation of sites in successive shells, this definition results in nearly featureless bulk-DOS data.

$\{p, q\}$	Conder Index	Number of Vertices
{7, 3}	C364.1	364
{7, 3}	C364.2	364
{7, 3}	C364.6	364
{8, 3}	C1632.2	1632
{8, 3}	C1632.6	1632
{8, 3}	C2048.23	2048
{8, 3}	C2048.24	2048
{8, 3}	C2048.25	2048
{10, 3}	C1440.4	1440
{10, 3}	C1440.6	1440
{10, 3}	C1680.2	1680
{10, 3}	C1920.3	1920

TABLE S3. **Regular maps used.** The regular maps used in this work are available in the online database provided by Conder [8], labelled type 2^1.

for comparison with a purely bulk theory. We remark that one can opt for an alternative definition of the bulk region to obtain different bulk-DOS results. For example, defining the boundary region as the outermost two shells gives a smaller bulk region. As shown in Supplementary Fig. S3, this definition improves the agreement in lattices {7, 3} and {8, 3} but generates nearly featureless bulk-DOS for lattices {10, 3}, {8, 4}, and {10, 5}. For the {7,3} lattice, the 5-shell flake features only 847 sites, which corresponds to approximately 15 unit cells. To confirm the prediction from HBT for larger systems, we tested a larger 6-shell {7,3}-flake with 2240 sites, corresponding to approximately 40 unit cells, and verified that the result for the bulk-DOS is indistinguishable when plotted against the 5-shell result shown in Fig. S2. This gives us confidence that the relatively small flake for {7,3} is a good testbed for the larger system.

Supplementary Discussion: HBT vs. Hyperbolic Regular Maps

Finite-sized hyperbolic lattices with periodic boundary condition are ideal for investigating bulk properties. While it is straightforward to construct periodic Euclidean lattices, periodic hyperbolic lattices can only exist on high-genus surfaces. Regular graphs of $\{p, q\}$ type on hyperbolic surfaces that preserve all local point-group symmetries are called *regular map* in graph theory. We obtain the adjacency matrices of several $\{p, 3\}$ regular maps from existing mathematical literature (see Table S3). Sizeable regular maps of {8, 4} and {10, 5} lattices are currently unavailable and thus omitted from our analysis. We then compute the eigenvalues and compare the corresponding DOS with predictions made by Bloch-wave Hamiltonians. As shown in Fig. S4 the comparison demonstrates close agreement with the exception of additional finite-size-induced gaps in the DOS of regular maps due to the finite number of vertices in the regular map. On lattice {8, 3}, the agreement is nearly exact.

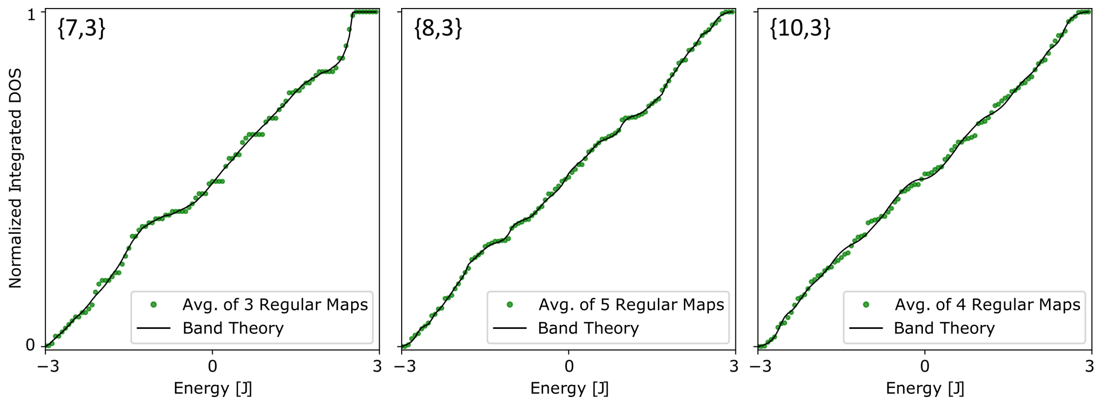

FIG. S4. **HBT vs. Hyperbolic Regular Maps**. We identified several regular maps for lattices {7, 3}, {8, 3}, and {10, 3} from the online database of Conder [8]. These regular maps are finite and boundary-less hyperbolic lattices embedded into high-genus surfaces. We compare the DOS obtained from their eigenvalues to band-theoretical predictions computed from Bloch-wave Hamiltonians. The comparison shows close agreement with the exception of additional finite-size-induced gaps in the DOS of regular maps. We average over several regular maps as they tend to have (likely accidental) degeneracies and finite-sized gaps in the energy spectra that we do not expect to represent the behavior of the infinite lattice. However, the agreement between HBT and each individual regular map is of comparable quality to the data shown here.

Supplementary Discussion: HBT vs. Higher-dimensional Euclidean lattices

By replacing the Bravais lattice of a hyperbolic $\{p, q\}$ lattice with a Euclidean $2g$-dimensional lattice, while keeping the unit cell unchanged, it is possible to create graphs where HBT is *exact*. Moreover, these lattices can be compactified to a torus to create finite periodic graphs. We will call these graphs $2g$-dimensional Euclidean lattices in the following. If the corresponding graph has $\mathcal{N}$ sites, then the eigenvalues of the $\mathcal{N} \times \mathcal{N}$ adjacency matrix, which are the single-particle states of the tight-binding Hamiltonian, are given exactly by a set of eigenvalues $\varepsilon_i = \varepsilon(\mathbf{k}_i)$, where $\varepsilon(\mathbf{k})$ are eigenvalues of the Bloch-wave Hamiltonian $H_{\{p,q\}}(\mathbf{k})$ and $\mathbf{k}_i, i = 1, \dots, \mathcal{N}$, is a set of known quantized momenta.

The existence of Abelian clusters of hyperbolic lattices, where all eigenstates transform under one-dimensional representations, was identified in Ref. [3]. In the latter work, they have been derived from quotients of the Fuchsian translation group by its normal subgroups. In the bottom-up-approach discussed in this supplementary section, we create a large Abelian cluster as a $2g$-dimensional Euclidean lattice, but we do not know if it always corresponds to an actual regular map or normal subgroup of hyperbolic lattices. In some instances, such as the $\{8, 3\}$ lattice, we will show that the Euclidean four-dimensional models are isomorphic to known regular maps. The good agreement between HBT and regular maps, which confirms the importance of Bloch wave theory for hyperbolic matter, strikes us as a solid motivation to study higher-dimensional Euclidean lattices in more detail in the future. They combine the characteristic higher-dimensional Brillouin zone of hyperbolic space with the usual easy access to all eigenstates in Euclidean band theory, hence lend themselves to simpler calculations of observables in hyperbolic matter.

To construct the higher-dimensional Euclidean lattices, we start from a $\{p, q\}$ lattice with unit dell $D = \{z_1, \dots, z_N\}$ with n sites and a $2g$-dimensional Bravais lattice. We specify the adjacency matrix (A_{ij}) through determining all nearest-neighbor bonds (i, j) with $A_{ij} = 1$. Note that the higher-dimensional Euclidean lattice is an undirected graph, so no complex phase factors appear in the Hamiltonian, only 1s and 0s. Any lattice site v_i is uniquely determined by the site in the unit cell z_n and the Bravais lattice vectors $\vec{\sigma} = (\sigma_1, \dots, \sigma_{2g}) \in \mathbb{N}^{2g}$. We write

$$v_i := z_i(\vec{\sigma}) \tag{S23}$$

in the following. We write $i \leftrightarrow j$ if sites v_i and v_j are nearest neighbors and $A_{ij} = 1$. We define $\hat{1} = (1, 0, 0, \dots, 0)$ and similarly $\hat{\mu}$ for $\mu = 1, \dots, 2g$. We use L_μ sites in each direction μ of the $2g$-dimensional lattice and impose periodic boundary conditions so that $\sigma_\mu + L_\mu = \sigma_\mu$ for each μ, hence $\sigma_\mu \in \{1, \dots, L\}$. The quantization of each momentum component appearing in the eigenvalues $\varepsilon(\mathbf{k}_i)$ is then given by

$$k_\mu = \frac{2\pi}{L_\mu} n_\mu, \; n_\mu = 0, \dots, L_\mu - 1, \tag{S24}$$

with $\mathbf{k}_i = (k_1, \dots, k_{2g})$. For all of the following lattices we confirm that the eigenvalues $\varepsilon_i = \varepsilon(\mathbf{k}_i)$ of the $\mathcal{N} \times \mathcal{N}$ adjacency matrix on the higher-dimensional Euclidean lattice agrees exactly with the prediction from $H_{\{p,q\}}(\mathbf{k})$ under

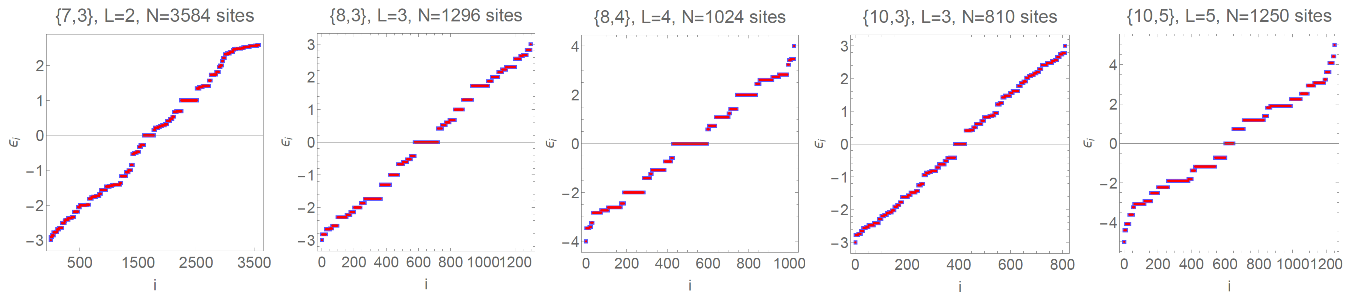

FIG. S5. **HBT vs. Higher-dimensional Euclidean lattices**. The eigenvalues on graphs obtained from higher-dimensional Euclidean lattices (blue) agree exactly with the predictions from HBT (red). We plot the eigenvalues ε_i (in units of J) vs. $i = 1, \ldots, \mathcal{N}$, where $\mathcal{N}$ is the number of vertices on the graph. Each plot is labelled by the $\{p, q\}$ lattice that is approximated through its unit cell in a Euclidean lattice of dimension $2g$, the number of lattice points in each Euclidean direction, L, and the number of sites given by $\mathcal{N} = NL^{2g}$, where N is the number of sites in the unit cell.

the quantization condition from Eq. (S24), see Fig. S5.

$\{8,3\}$-*lattice.* The unit cell has 16 sites $\{z_1, \ldots, z_{16}\}$ and momentum space is four-dimensional. The Euclidean Bravais lattices is given by $\vec{\sigma} = (\sigma_1, \sigma_2, \sigma_3, \sigma_4)$. The adjacency matrix is determined by the bonds

$$\begin{aligned}
&z_1(\vec{\sigma}) \leftrightarrow z_2(\vec{\sigma}),\ z_1(\vec{\sigma}) \leftrightarrow z_8(\vec{\sigma}),\ z_1(\vec{\sigma}) \leftrightarrow z_9(\vec{\sigma}),\ z_2(\vec{\sigma}) \leftrightarrow z_3(\vec{\sigma}),\ z_2(\vec{\sigma}) \leftrightarrow z_{10}(\vec{\sigma}),\ z_3(\vec{\sigma}) \leftrightarrow z_4(\vec{\sigma}),\ z_3(\vec{\sigma}) \leftrightarrow z_{11}(\vec{\sigma}),\\
&z_4(\vec{\sigma}) \leftrightarrow z_5(\vec{\sigma}),\ z_4(\vec{\sigma}) \leftrightarrow z_{12}(\vec{\sigma}),\ z_5(\vec{\sigma}) \leftrightarrow z_6(\vec{\sigma}),\ z_5(\vec{\sigma}) \leftrightarrow z_{13}(\vec{\sigma}),\ z_6(\vec{\sigma}) \leftrightarrow z_7(\vec{\sigma}),\ z_6(\vec{\sigma}) \leftrightarrow z_{14}(\vec{\sigma}),\ z_7(\vec{\sigma}) \leftrightarrow z_8(\vec{\sigma}),\\
&z_7(\vec{\sigma}) \leftrightarrow z_{15}(\vec{\sigma}),\ z_8(\vec{\sigma}) \leftrightarrow z_{16}(\vec{\sigma}),\ z_9(\vec{\sigma}) \leftrightarrow z_{12}(\vec{\sigma}+\hat{1}),\ z_9(\vec{\sigma}) \leftrightarrow z_{14}(\vec{\sigma}+\hat{2}),\ z_{10}(\vec{\sigma}) \leftrightarrow z_{13}(\vec{\sigma}+\hat{2}),\\
&z_{10}(\vec{\sigma}) \leftrightarrow z_{15}(\vec{\sigma}+\hat{3}),\ z_{11}(\vec{\sigma}) \leftrightarrow z_{14}(\vec{\sigma}+\hat{2}),\ z_{11}(\vec{\sigma}) \leftrightarrow z_{16}(\vec{\sigma}+\hat{4}),\ z_{12}(\vec{\sigma}) \leftrightarrow z_{15}(\vec{\sigma}+\hat{4}),\ z_{16}(\vec{\sigma}) \leftrightarrow z_{13}(\vec{\sigma}+\hat{1}),
\end{aligned} \tag{S25}$$

see Eq. (S16). The number of sites is $\mathcal{N} = 16 \cdot L_1 L_2 L_3 L_4$. For $L_1 = L_2 = L_3 = L_4 = L$ we have $\mathcal{N} = 16L^4$. We confirmed that the graphs defined on the Euclidean lattices with $L = 1, 2, 3$ ($\mathcal{N} = 16, 256, 1296$) are isomorphic to the regular maps with Condor index C16.1 (Moebius-Kantor-Graph), C256.4, C1296.1. For $L = 4$, we obtain $\mathcal{N} = 4096$, which is larger than the largest regular map available for comparison.

$\{8,4\}$-*lattice.* The unit cell has four sites $\{z_1, \ldots, z_4\}$ and momentum space is four-dimensional. The adjacency matrix is constructed from the bonds

$$\begin{aligned}
&z_1(\vec{\sigma}) \leftrightarrow z_2(\vec{\sigma}),\ z_1(\vec{\sigma}) \leftrightarrow z_2(\vec{\sigma}+\hat{1}-\hat{2}),\ z_1(\vec{\sigma}) \leftrightarrow z_4(\vec{\sigma}+\hat{1}),\ z_4(\vec{\sigma}) \leftrightarrow z_1(\vec{\sigma}+\hat{4}),\\
&z_2(\vec{\sigma}) \leftrightarrow z_3(\vec{\sigma}),\ z_2(\vec{\sigma}) \leftrightarrow z_3(\vec{\sigma}+\hat{2}-\hat{3}),\ z_3(\vec{\sigma}) \leftrightarrow z_4(\vec{\sigma}),\ z_3(\vec{\sigma}) \leftrightarrow z_4(\vec{\sigma}+\hat{3}-\hat{4}),
\end{aligned} \tag{S26}$$

see Eq. (S15). The number of sites is $\mathcal{N} = 4 \cdot L_1 L_2 L_3 L_4$.

$\{10,3\}$-*lattice.* The unit cell has ten sites $\{z_1, \ldots, z_{10}\}$ and the Euclidean Bravais lattice is four-dimensional. The direction associated to the fifth generator γ_5 of the $\{10,5\}$ Bravais lattice is implemented via $k_5 = -k_1 + k_2 - K_3 + k_4$. The adjacency matrix consists of the bonds

$$\begin{aligned}
&z_1(\vec{\sigma}) \leftrightarrow z_2(\vec{\sigma}),\ z_1(\vec{\sigma}) \leftrightarrow z_{10}(\vec{\sigma}),\ z_2(\vec{\sigma}) \leftrightarrow z_3(\vec{\sigma}),\ z_3(\vec{\sigma}) \leftrightarrow z_4(\vec{\sigma}),\ z_4(\vec{\sigma}) \leftrightarrow z_5(\vec{\sigma}),\ z_5(\vec{\sigma}) \leftrightarrow z_6(\vec{\sigma}),\ z_6(\vec{\sigma}) \leftrightarrow z_7(\vec{\sigma}),\\
&z_7(\vec{\sigma}) \leftrightarrow z_8(\vec{\sigma}),\ z_8(\vec{\sigma}) \leftrightarrow z_9(\vec{\sigma}),\ z_9(\vec{\sigma}) \leftrightarrow z_{10}(\vec{\sigma}),\ z_1(\vec{\sigma}) \leftrightarrow z_6(\vec{\sigma}+\hat{1}),\ z_2(\vec{\sigma}) \leftrightarrow z_7(\vec{\sigma}+\hat{2}),\ z_3(\vec{\sigma}) \leftrightarrow z_8(\vec{\sigma}+\hat{3}),\\
&z_4(\vec{\sigma}) \leftrightarrow z_9(\vec{\sigma}+\hat{4}),\ z_5(\vec{\sigma}) \leftrightarrow z_{10}(\vec{\sigma}-\hat{1}+\hat{2}-\hat{3}+\hat{4}),
\end{aligned} \tag{S27}$$

see Eq. (S17). The number of sites is $\mathcal{N} = 10 \cdot L_1 L_2 L_3 L_4$.

$\{10,5\}$-*lattice.* The unit cell has two sites $\{z_1, z_2\}$ and the four-dimensional momentum space is implemented on the Euclidean lattice as in the $\{10,3\}$-case above. The adjacency matrix is given by the bonds

$$\begin{aligned}
&z_1(\vec{\sigma}) \leftrightarrow z_2(\vec{\sigma}),\ z_1(\vec{\sigma}) \leftrightarrow z_2(\vec{\sigma}+\hat{1}-\hat{2}),\ z_1(\vec{\sigma}) \leftrightarrow z_2(\vec{\sigma}+\hat{2}-\hat{3}),\\
&z_1(\vec{\sigma}) \leftrightarrow z_2(\vec{\sigma}+\hat{1}-\hat{3}+\hat{4}),\ z_1(\vec{\sigma}) \leftrightarrow z_2(\vec{\sigma}+\hat{2}-\hat{3}-\hat{3}+\hat{4}),
\end{aligned} \tag{S28}$$

see Eq. (S13). The number of sites is $\mathcal{N} = 2 \cdot L_1 L_2 L_3 L_4$.

$\{7,3\}$-*lattice.* The unit cell has 56 sites $\{z_1, \ldots, z_{56}\}$ and momentum space is six-dimensional, $\vec{\sigma} = (\sigma_1, \ldots, \sigma_6)$. The seventh generator γ_7 of the $\{14,7\}$ Bravais lattice is implemented through $k_7 = -k_1 + k_2 - k_3 + k_4 - k_5 + k_6$. The adjacency matrix of the Euclidean higher-dimensional lattice is constructed in the same way as the previous example, starting from the bonds given in Table S1. The number of sites is $\mathcal{N} = 56 \cdot L_1 L_2 L_3 L_4 L_5 L_6$.

SUPPLEMENTARY SECTION S IV NODAL REGION AND DIRAC HAMILTONIAN OF HYPERBOLIC GRAPHENE

The Bloch-wave Hamiltonian of hyperbolic graphene can be written as

$$H_{\{10,5\}}(\mathbf{k}) = d_x(\mathbf{k})\sigma_x + d_y(\mathbf{k})\sigma_y, \tag{S29}$$

where σ_i are the Pauli matrices and

$$d_x(\mathbf{k}) = -1 - \sum_{\mu=1}^{4} \cos(k_\mu) \tag{S30}$$

$$d_y(\mathbf{k}) = -\sum_{\mu=1}^{4} \sin(k_\mu) \tag{S31}$$

with hopping amplitude J set to 1. The energy bands are

$$\varepsilon_\pm(\mathbf{k}) = \pm\sqrt{d_x(\mathbf{k})^2 + d_y(\mathbf{k})^2}. \tag{S32}$$

The nodal (or band-touching) region thus satisfies

$$d_x(\mathbf{k}) = 0 \text{ and } d_y(\mathbf{k}) = 0. \tag{S33}$$

We eliminate k_4 using $\cos^2 k_4 + \sin^2 k_4 = 1$ and obtain the following equation:

$$\left(1 + \sum_{\mu=1}^{3} \cos(k_\mu)\right)^2 + \left(\sum_{\mu=1}^{3} \sin(k_\mu)\right)^2 = 1.$$

Having three variables, this equation defines a two-dimensional surface, which is the nodal region $\mathcal{S}$ as projected onto the three-dimensional hyperplane (k_1, k_2, k_3) (see Fig. 4b of the main text).

We now show that $H_{\{10,5\}}(\mathbf{k})$ is approximated by a Dirac Hamiltonian at every node $\mathbf{Q} \in \mathcal{S}$. Expanding $d_x(\mathbf{k})$ and $d_x(\mathbf{k})$ at $\mathbf{k} = \mathbf{Q} + \mathbf{q}$ for small $\mathbf{q}$ gives

$$d_x(\mathbf{Q}+\mathbf{q}) \sim d_x(\mathbf{Q}) + \sum_{\mu=1}^{4} \left.\frac{\partial d_x}{\partial k_\mu}\right|_{\mathbf{Q}} q_\mu + \mathcal{O}(q^2) = \sum_{\mu=1}^{4} \sin(Q_\mu) q_\mu + \mathcal{O}(q^2) \tag{S34}$$

$$d_y(\mathbf{Q}+\mathbf{q}) \sim d_y(\mathbf{Q}) + \sum_{\mu=1}^{4} \left.\frac{\partial d_y}{\partial k_\mu}\right|_{\mathbf{Q}} q_\mu + \mathcal{O}(q^2) = -\sum_{\mu=1}^{4} \cos(Q_\mu) q_\mu + \mathcal{O}(q^2) \tag{S35}$$

In the basis of vectors

$$\mathbf{u}(\mathbf{Q}) = \sum_{\mu=1}^{4} \sin(Q_\mu)\mathbf{e}_\mu \text{ and } \mathbf{v}(\mathbf{Q}) = \sum_{\mu=1}^{4} \cos(Q_\mu)\mathbf{e}_\mu, \tag{S36}$$

where $\mathbf{e}_\mu$ are the standard Cartesian unit vectors, the Hamiltonian near $\mathbf{Q}$,

$$h_{\text{eff}}^{\mathbf{Q}}(\mathbf{q}) = \sigma_x \mathbf{q} \cdot \mathbf{u}(\mathbf{Q}) - \sigma_y \mathbf{q} \cdot \mathbf{v}(\mathbf{Q}) + \mathcal{O}(q^2), \tag{S37}$$

describes relativistic Dirac particles with anisotropic velocities given by $|\mathbf{u}(\mathbf{Q})|$ and $|\mathbf{v}(\mathbf{Q})|$. We confirmed that $\mathbf{u}(\mathbf{Q})$ and $\mathbf{v}(\mathbf{Q})$ are nonzero and linearly independent for $\mathbf{Q} \in \mathcal{S}$.

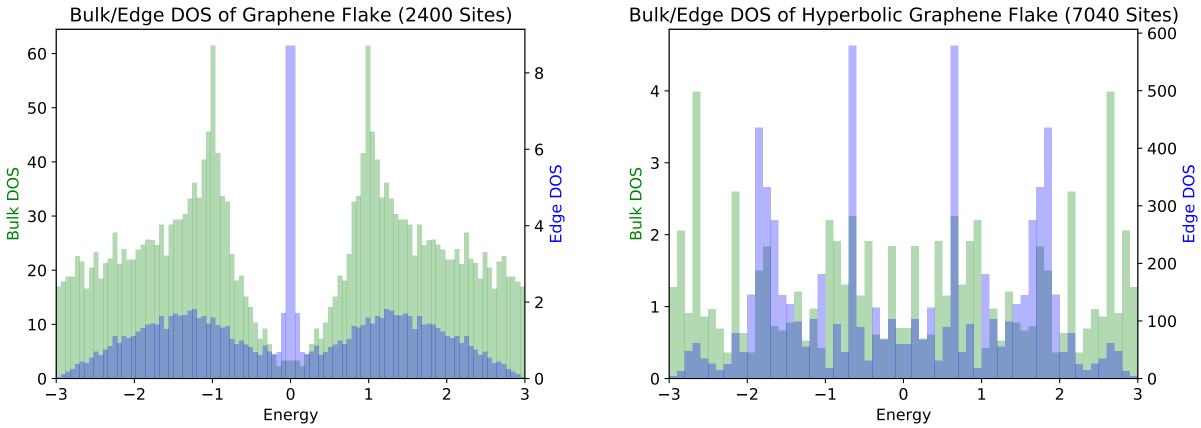

FIG. S6. **Bulk-boundary correspondence.** *Left.* We compute the bulk-DOS and edge-DOS on a finite $\{6,3\}$-flake with 2400 sites. While there is a reduced bulk-DOS at zero energy $E = 0$, due to the system being semi-metallic, a sharp peak of edge-DOS is visible at $E = 0$, corresponding to a topological boundary mode. *Right.* A similar analysis of a $\{10,5\}$-flake, i.e. hyperbolic graphene, with 7040 sites does not yield the same pattern. While pronounced peaks of the edge-DOS are visible, they do not appear in energy regions of reduced bulk-DOS, and so their topological nature cannot be inferred from this analysis.

SUPPLEMENTARY SECTION S V BULK-BOUNDARY CORRESPONDENCE

In its idealized version of fermions hopping on a honeycomb lattice, semi-metallic graphene is a topological semimetal with zero-energy boundary states [9]. The reason for this is that for any one-dimensional cut through the two-dimensional Brillouin zone (avoiding a Dirac point), the Bloch wave Hamiltonian realizes a one-dimensional topological insulator in class AIII with protected boundary states in position space. We confirm this behavior in a numerical diagonalization of a $\{6,3\}$ flake: while the bulk DOS is small near zero energy, the edge DOS (defined as the difference between total BOS and bulk DOS) shows a pronounced peak at zero energy, see Fig. S6 Note that for this argument to work, the equality of dimension of position and momentum space are crucial.

The bulk topology of hyperbolic graphene is the four-dimensional analogue of graphene, as demonstrated by the π Berry phase around each Dirac node in the band-touching manifold. However, a similar theoretical construction of cuts in momentum space remains inconclusive, since position and momentum space have different dimensions. While first studies on the topological properties of hyperbolic lattices have appeared recently, the interplay between position and momentum space invariants remains an open problem.

We address the presence of boundary states in hyperbolic graphene with an unbiased numerical analysis. By using a finite-sized $\{10,5\}$ flake with 7040 sites, we compare bulk DOS and edge DOS, see Fig. S6. We observe that there is no pronounced peak of edge states at zero energy. While some energy ranges are strongly populated with edge states, these regimes do not coincide with regions of small bulk DOS so that their topological interpretation is questionable. On the other hand, we cannot fully exclude the possibility that topological boundary modes are present, as they might be obscured by the inherent inaccuracy in the separation of total DOS into bulk and edge contributions. This is particularly true for $\{10,5\}$-flakes, which have an enormous fraction of boundary sites.

SUPPLEMENTARY SECTION S VI FLOQUET BAND GAPS IN HYPERBOLIC GRAPHENE

In this supplementary section, we briefly describe the Floquet theory formalism (see for example Ref. [10]). Then we apply it to the Bloch-wave Hamiltonian of hyperbolic graphene with time-periodic momentum components. We compute the quasi-energy spectrum of the resulting Floquet system and observe gap opening at the nodal region of hyperbolic graphene. Moreover, the induced gap size varies over the nodal region.

Supplementary Discussion: Brief Review of Floquet Formalism

Consider a quantum system with periodic time-dependence such that the Hamiltonian follows $H(t+T) = H(t)$, where T is the period. Analogous to the spatial translation symmetry of a crystal, the system respects a discrete translation symmetry in time. The "translation operator" is the stroboscopic evolution operator, i.e. the evolution operator over one period,

$$U = \mathcal{T} e^{-i\int_0^T H(t)\,dt}. \tag{S38}$$

Here $\mathcal{T}$ is the time-ordering operator. Note that U has no time-dependence. The stationary eigenstates of U are called the *Floquet states* and form a complete basis for the solutions to the Schrödinger equation

$$i\frac{d}{dt}|\psi(t)\rangle = H(t)|\psi(t)\rangle. \tag{S39}$$

Unless the time-dependence of $H(t)$ is very simple (e.g. a step function), it is generally difficult to solve for U from Eq. (S38) and diagonalize it. The problem is much simpler in the frequency space, as shown below.

Analogous to a Bloch state which is the product of a plane wave and a spatially periodic function, every Floquet state is of the form (by Floquet's theorem)

$$|\psi(t)\rangle = e^{-i\varepsilon t}|u(t)\rangle, \tag{S40}$$

where $|u(t+T)\rangle = |u(t)\rangle$ and ε is called the quasi-energy of the Floquet state. Plugging this ansatz into the Schrödinger equation gives

$$(\varepsilon + i\frac{d}{dt})|u(t)\rangle = H(t)|u(t)\rangle. \tag{S41}$$

Because of the periodicity of $H(t)$ and $u(t)$, we can Fourier transform this equation to frequency space by Fourier decomposition

$$|u(t)\rangle = \sum_n e^{-in\omega t}|u^{(n)}\rangle, \tag{S42}$$

$$H(t) = \sum_n e^{-in\omega t}H^{(n)}, \tag{S43}$$

where $\omega = 2\pi/T$. Plugging this into Eq. (S41) yields

$$(\varepsilon + n\omega)|u^{(n)}\rangle = \sum_m H^{(n-m)}|u^{(m)}\rangle. \tag{S44}$$

There are infinitely many equations, but in general a truncated set of Fourier harmonics is sufficient to approximate the Floquet states and their quasi-energies to arbitrary accuracy.

Supplementary Discussion: Time-Periodic Hyperbolic Graphene

Given the Bloch-wave Hamiltonian of hyperbolic graphene in Eq. (4), we add time-periodic terms to the momentum components

$$H_{\{10,5\}}(\mathbf{k},t) = -J\begin{pmatrix} 0 & 1+\sum\limits_{\mu=1}^{4} e^{\mathrm{i}(k_\mu - A\sin(\omega t+\varphi_\mu))} \\ \text{c.c.} & 0 \end{pmatrix}, \tag{S45}$$

where J is the nearest neighbour hopping amplitude, A is the driving amplitude, ω is the frequency, and $\varphi_1, ..., \varphi_4$ are phase shifts in the sinusoidal terms. This model is inspired by previous studies on irradiated graphene [11, 12], where the vector potential of a circularly polarized light, $\mathbf{a}(t) = a_0(\sin(\omega t), \cos(\omega t))$, modifies the momentum as $k_x \to k_x - ea_0\sin(\omega t)$ and $k_y \to k_y - ea_0\cos(\omega t)$.

To solve for the Floquet states, we compute the Fourier components of $H_{\{10,5\}}(\mathbf{k},t)$ as

$$H^{(m)}(\mathbf{k}) = \frac{1}{T}\int_0^T \mathrm{d}t\; e^{im\omega t} H_{\{10,5\}}(\mathbf{k},t) \tag{S46}$$

$$= -J\begin{pmatrix} 0 & \delta_{m0} + \mathcal{J}_{-m}(A)\sum_{\mu=1}^4 e^{\mathrm{i}(k_\mu - m\varphi_\mu)} \\ \delta_{m0} + \mathcal{J}_m(A)\sum_{\mu=1}^4 e^{-\mathrm{i}(k_\mu + m\varphi_\mu)} & 0 \end{pmatrix}, \tag{S47}$$

where we used

$$\frac{1}{T}\int_0^T \mathrm{d}t\; e^{\mathrm{i}m\omega t \pm A\sin(\omega t+\varphi)} = (\mp 1)^m e^{-\mathrm{i}m\varphi}\mathcal{J}_m(A) \tag{S48}$$

with $\mathcal{J}_m$ the Bessel function of the first kind. Note that in the limit $A \ll 1$, the integrals are proportional to $A^{|m|}$, so $H^{(m)} \sim \mathcal{O}(A^{|m|})$. We work in the limit of small driving amplitude A and keep terms up to $\mathcal{O}(A^2)$. Rewriting Eq. (S44) in matrix form gives

$$\begin{pmatrix} H^{(0)}-2\omega & H^{(-1)} & H^{(-2)} & 0 & 0 \\ H^{(1)} & H^{(0)}-\omega & H^{(-1)} & H^{(-2)} & 0 \\ H^{(2)} & H^{(1)} & H^{(0)} & H^{(-1)} & H^{(-2)} \\ 0 & H^{(2)} & H^{(1)} & H^{(0)}+\omega & H^{(-1)} \\ 0 & 0 & H^{(2)} & H^{(1)} & H^{(0)}+2\omega \end{pmatrix} \begin{pmatrix} |u^{(-2)}\rangle \\ |u^{(-1)}\rangle \\ |u^{(0)}\rangle \\ |u^{(1)}\rangle \\ |u^{(2)}\rangle \end{pmatrix} = E \begin{pmatrix} |u^{(-2)}\rangle \\ |u^{(-1)}\rangle \\ |u^{(0)}\rangle \\ |u^{(1)}\rangle \\ |u^{(2)}\rangle \end{pmatrix}, \tag{S49}$$

where we have truncated the matrix to only contain Fourier harmonics $-2 \le n \le 2$. This truncation does not affect the subsequent calculations using degenerate perturbation theory. The unperturbed Hamiltonian is

$$H_0 = \begin{pmatrix} \begin{pmatrix} -2\omega & \varepsilon(\mathbf{k}) \\ \varepsilon(\mathbf{k})^* & -2\omega \end{pmatrix} & 0 & 0 & 0 & 0 \\ 0 & \begin{pmatrix} -\omega & \varepsilon(\mathbf{k}) \\ \varepsilon(\mathbf{k})^* & -\omega \end{pmatrix} & 0 & 0 & 0 \\ 0 & 0 & \begin{pmatrix} 0 & \varepsilon(\mathbf{k}) \\ \varepsilon(\mathbf{k})^* & 0 \end{pmatrix} & 0 & 0 \\ 0 & 0 & 0 & \begin{pmatrix} \omega & \varepsilon(\mathbf{k}) \\ \varepsilon(\mathbf{k})^* & \omega \end{pmatrix} & 0 \\ 0 & 0 & 0 & 0 & \begin{pmatrix} 2\omega & \varepsilon(\mathbf{k}) \\ \varepsilon(\mathbf{k})^* & 2\omega \end{pmatrix} \end{pmatrix}, \tag{S50}$$

where

$$\varepsilon(\mathbf{k}) = -J(1 + \sum_{\mu=1}^4 e^{\mathrm{i}k_\mu}), \tag{S51}$$

and the perturbation is

$$H_1 = \begin{pmatrix} \begin{pmatrix} 0 & a(\mathbf{k}) \\ a(\mathbf{k})^* & 0 \end{pmatrix} & H^{(-1)} & H^{(-2)} & 0 & 0 \\ H^{(1)} & \begin{pmatrix} 0 & a(\mathbf{k}) \\ a(\mathbf{k})^* & 0 \end{pmatrix} & H^{(-1)} & H^{(-2)} & 0 \\ H^{(2)} & H^{(1)} & \begin{pmatrix} 0 & a(\mathbf{k}) \\ a(\mathbf{k})^* & 0 \end{pmatrix} & H^{(-1)} & H^{(-2)} \\ 0 & H^{(2)} & H^{(1)} & \begin{pmatrix} 0 & a(\mathbf{k}) \\ a(\mathbf{k})^* & 0 \end{pmatrix} & H^{(-1)} \\ 0 & 0 & H^{(2)} & H^{(1)} & \begin{pmatrix} 0 & a(\mathbf{k}) \\ a(\mathbf{k})^* & 0 \end{pmatrix} \end{pmatrix}, \tag{S52}$$

where

$$a(\mathbf{k}) = -J(\mathcal{J}_0(A)-1)\sum_{\mu=1}^{4} e^{\mathrm{i}k_\mu} \approx \frac{JA^2}{4}\sum_{\mu=1}^{4} e^{\mathrm{i}k_\mu} + \mathcal{O}(A^4). \tag{S53}$$

The unperturbed quasi-energy spectrum consists of many identical copies of the $H_{\{10,5\}}$ energy spectrum, $\pm|\varepsilon(\mathbf{k})|$, shifted by $n\omega$. For $\mathbf{k}$ near the nodal region $\mathcal{S}$, each pair of levels are nearly degenerate in comparison to their separation ω from all the other bands, i.e. $|\varepsilon(\mathbf{k})| \ll \omega$. In this limit we can apply degenerate perturbation theory. Since the middle two bands ($n = 0$) are the best approximation of the quasi-energy spectrum (the other bands are more distorted copies [10]), we will focus on the middle two bands and compute the energy-splitting at the nodal region. The effective Hamiltonian describing the energy splitting is [13]

$$H_{\text{eff}} = PH_0P + PH_1P + PH_1\frac{1-P}{E_0 - H_0}H_1P, \tag{S54}$$

where $E_0 = 0$ is the energy at the nodal region and P is the projection operator onto the middle two bands given by

$$P = \begin{pmatrix} \begin{pmatrix} 0 & 0 \\ 0 & 0 \end{pmatrix} & 0 & 0 & 0 & 0 \\ 0 & \begin{pmatrix} 0 & 0 \\ 0 & 0 \end{pmatrix} & 0 & 0 & 0 \\ 0 & 0 & \begin{pmatrix} 1 & 0 \\ 0 & 1 \end{pmatrix} & 0 & 0 \\ 0 & 0 & 0 & \begin{pmatrix} 0 & 0 \\ 0 & 0 \end{pmatrix} & 0 \\ 0 & 0 & 0 & 0 & \begin{pmatrix} 0 & 0 \\ 0 & 0 \end{pmatrix} \end{pmatrix}. \tag{S55}$$

Plugging in H_0 and H_1 yields

$$H_{\text{eff}}(\mathbf{k}) = -J\begin{pmatrix} 0 & 1 + \mathcal{J}_0(A)\sum_{\mu=1}^{4} e^{\mathrm{i}k_\mu} \\ \text{c.c.} & 0 \end{pmatrix} + \Delta(\mathbf{k})\sigma_z + \mathcal{O}(A^4) \tag{S56}$$

with

$$\Delta(\mathbf{k}) = \frac{J^2A^2}{2\omega}\sum_{\mu=1}\sum_{\substack{\nu=1\\ \nu\neq\mu}} \sin(k_\mu - k_\nu)\sin(\varphi_\mu - \varphi_\nu). \tag{S57}$$

The first term in Eq. (S56) causes a small, smooth deformation of the nodal region $\mathcal{S}$ in the Brillouin zone, without gapping out any node. On the other hand, the second term clearly introduces a gap of size $2|\Delta(\mathbf{k})|$. The gap size varies across the nodal surface, in contrast to irradiated graphene where both Dirac nodes are gapped out by the same magnitude. Moreover, for a generic selection of phase shifts $\{\varphi_1, ..., \varphi_4\}$, $\Delta(\mathbf{k})$ is zero for some $\mathbf{k} \in \mathcal{S}$, which means that a part of the nodal region remains nearly gapless (up to $\mathcal{O}(A^4)$). To see this, let us recall that the nodal region is solved by setting $|\varepsilon(\mathbf{k})| = 0$. Since $\varepsilon(\mathbf{k})$ is complex, this condition gives two equations: $\mathrm{Re}(\varepsilon(\mathbf{k})) = 0$ and $\mathrm{Im}(\varepsilon(\mathbf{k})) = 0$. Further requiring that $\Delta(\mathbf{k}) = 0$ results in three independent equations. With four momentum components, the solution set is generally one-dimensional, implying a one-dimensional nearly gapless region.

We further investigate the non-uniform gap opening by exact diagonalization of Eq. (S49), choosing $\varphi_n = \pi n/2$. The results are shown in Fig. 4f of the main text and Supplementary Fig. S7. Figure 4f visualizes the band structure in the two-dimensional subspace $(k_3, k_4) = (2\pi/3, -\pi/3)$. Here we exaggerate the gap opening by choosing non-perturbative parameters $A = 0.8$, $\omega = 6$, and $J = 1$. Supplementary Fig. S7 plots the gap size along two loops in the Brillouin zone. Here $A = 0.1$ and $\omega = 10$ are in the perturbative limit. In principle, the nearly gapless nodes can be in fact gapped by high-order terms in A, but such a small gap may not be resolvable in experiments.

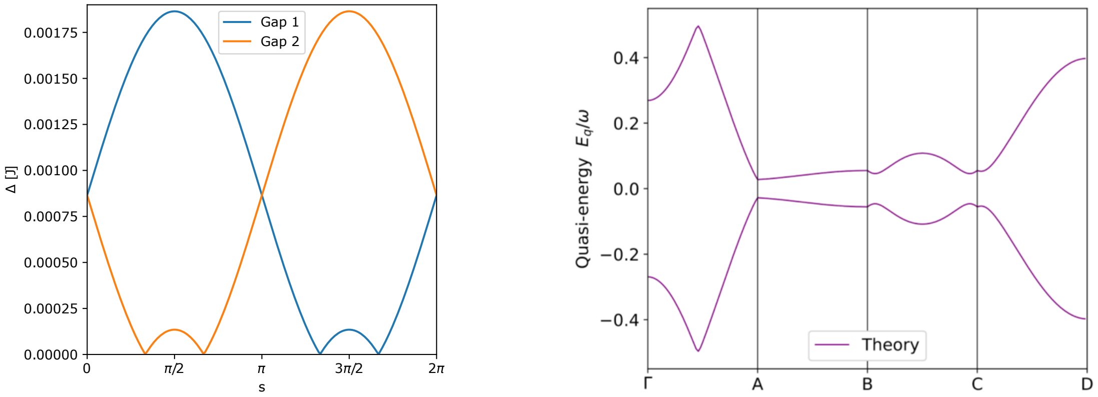

FIG. S7. **Floquet-driven hyperbolic graphene.** *Left.* We demonstrate how the gap size varies drastically over the nodal surface by keeping track of the gap opening at the two nodes in the momentum planes $(k_3, k_4) = (s, s+\pi)$ with $s \in [0, 2\pi]$. Notably, one node remains nearly gapless (up to $\mathcal{O}(A^4)$) at $s = \pi/3, 2\pi/3, 4\pi/3, 5\pi/3$. Here $A = 0.1$, $\omega = 10$, and $J = 1$. *Right.* Gap-opening due to the Floquet drive along the continuous line in momentum space that is shown in Fig. 4d without Floquet drive. Note that for the non-driven system, there is an extended gapless region from A to B, and a band-crossing point at C.

SUPPLEMENTARY SECTION S VII DERIVATION OF THE CIRCUIT LAPLACIAN OF THE PHASE ELEMENT

In the following, we derive the circuit Laplacian of the complex phase element from the main text (see Fig. S8). Assuming that no current is flowing into any of the inputs of the multipliers, for the currents flowing into the phase element, we have

$$I_1 = -I_{Z_2} - I_{R_2}, \tag{S58}$$
$$I_2 = -I_{Z_1} - I_{R_1}. \tag{S59}$$

Using the transfer function of the multipliers $W = \frac{(X_1 - X_2)\cdot(Y_1 - Y_2)}{10\,\mathrm{V}} + Z$ and noting that the Z input was used to compensate for output offsets and therefore in this derivation can be set to zero, the currents on the right hand sides can be rewritten as

$$I_1 = -\frac{1}{Z_2}\left(\frac{X_{1,2}\,Y_{1,2}}{10\,\mathrm{V}} - V_1\right) - \frac{1}{R_2}\left(\frac{X_{2,2}\,Y_{2,2}}{10\,\mathrm{V}} - V_1\right), \tag{S60}$$
$$I_2 = -\frac{1}{Z_1}\left(\frac{X_{1,1}\,Y_{1,1}}{10\,\mathrm{V}} - V_2\right) - \frac{1}{R_1}\left(\frac{X_{2,1}\,Y_{2,1}}{10\,\mathrm{V}} - V_2\right). \tag{S61}$$

The two indices on X and Y indicate position as shown in Fig. S8. Plugging in the voltages which are connected to the inputs of the multipliers yields

$$I_1 = -\frac{1}{Z_2}\left(\frac{V_a\,V_2}{10\,\mathrm{V}} - V_1\right) - \frac{1}{R_2}\left(\frac{-V_b\,V_2}{10\,\mathrm{V}} - V_1\right), \tag{S62}$$
$$I_2 = -\frac{1}{Z_1}\left(\frac{V_a\,V_1}{10\,\mathrm{V}} - V_2\right) - \frac{1}{R_1}\left(\frac{V_b\,V_1}{10\,\mathrm{V}} - V_2\right). \tag{S63}$$

Introduce the shorthand notation $a := \frac{V_a}{10\,\mathrm{V}}$ and $b := \frac{V_b}{10\,\mathrm{V}}$, so that

$$I_1 = -\left(\frac{a}{Z_2} - \frac{b}{R_2}\right)V_2 + \left(\frac{1}{Z_2} + \frac{1}{R_2}\right)V_1, \tag{S64}$$
$$I_2 = -\left(\frac{a}{Z_1} + \frac{b}{R_1}\right)V_1 + \left(\frac{1}{Z_1} + \frac{1}{R_1}\right)V_2, \tag{S65}$$

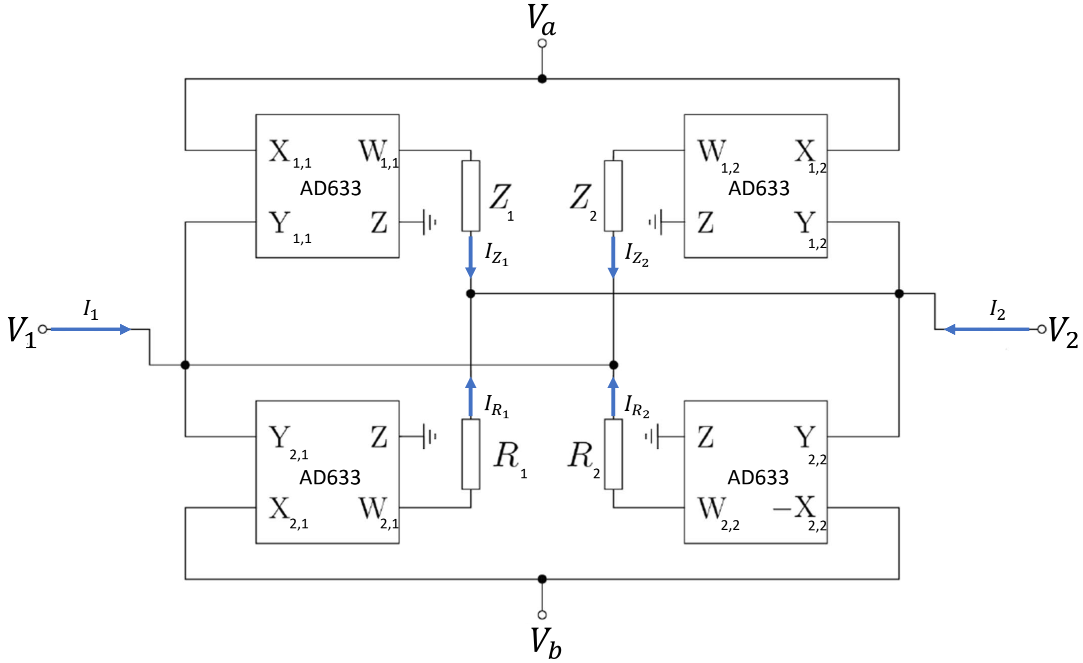

FIG. S8. **Complex-phase element (schematic).** Circuit diagram of the implementation of a boundary phase element using analog multipliers to enable continuous gain tuning.

or, as a matrix equation

$$\begin{pmatrix} I_1 \\ I_2 \end{pmatrix} = \begin{pmatrix} \frac{1}{Z_2} + \frac{1}{R_2} & -\left(\frac{a}{Z_2} - \frac{b}{R_2}\right) \\ -\left(\frac{a}{Z_1} + \frac{b}{R_1}\right) & \frac{1}{Z_1} + \frac{1}{R_1} \end{pmatrix} \begin{pmatrix} V_1 \\ V_2 \end{pmatrix}. \tag{S66}$$

Choosing $Z_1 = Z_2 =: Z$, $R := R_1 = R_2 = |Z|$ and $\mathrm{i}\, R = Z$, this equation becomes

$$\begin{pmatrix} I_1 \\ I_2 \end{pmatrix} = \frac{1}{Z} \begin{pmatrix} 1 + \mathrm{i} & -(a - \mathrm{i}b) \\ -(a + \mathrm{i}b) & 1 + \mathrm{i} \end{pmatrix} \begin{pmatrix} V_1 \\ V_2 \end{pmatrix}. \tag{S67}$$

If, as in our experimental realization of the phase element, the impedance is chosen to be an inductor with $Z = \mathrm{i}\omega L$, then $R = \omega L$ for the resistance. As a last step, the applied voltages V_a and V_b are chosen to be $10\,\mathrm{V}\,\cos(k)$ and $10\,\mathrm{V}\,\sin(k)$, respectively, with $k \in [0, 2\,\pi[$ which transforms the above matrix equation into the form of the main text:

$$\begin{pmatrix} I_1 \\ I_2 \end{pmatrix} = \frac{1}{\mathrm{i}\omega L} \begin{pmatrix} 1 + \mathrm{i} & -e^{-\mathrm{i}k} \\ -e^{\mathrm{i}k} & 1 + \mathrm{i} \end{pmatrix} \begin{pmatrix} V_1 \\ V_2 \end{pmatrix}. \tag{S68}$$

In the case of hyperbolic graphene, the unit cell consists of two nodes connected by an inductor of inductance L, with capacitances C to ground at each of the nodes. Therefore, the Laplacian of this unit cell reads

$$L_{\mathrm{UC}} = \begin{pmatrix} \mathrm{i}\omega C + \frac{1}{\mathrm{i}\omega L} & -\frac{1}{\mathrm{i}\omega L} \\ -\frac{1}{\mathrm{i}\omega L} & \mathrm{i}\omega C + \frac{1}{\mathrm{i}\omega L} \end{pmatrix}. \tag{S69}$$

Due to nonlinear behaviour of the multipliers for applied voltages at the upper boundary of the allowed input range, i.e. voltages near $10\,\mathrm{V}$, the magnitude of the impedances where cut in half by using two identical inductors in parallel and reducing the resistance with $R = \omega L$ still holding. Therefore the control voltages V_a and V_b can be operated in half of the input range, i.e. $\pm 5\,\mathrm{V}$, leading to the same behaviour of the phase element with doubled diagonal entries. The full Laplacian of four phase elements connected to the unit cell is the sum of the above Laplacians,

$$L_{\mathrm{full}} = \begin{pmatrix} \mathrm{i}\omega C + \frac{9}{\mathrm{i}\omega L} + \frac{\mathrm{i}}{\mathrm{i}\omega L} & -\frac{1}{\mathrm{i}\omega L}\left(1 + \sum_{q=1}^{4} e^{-\mathrm{i}k_q}\right) \\ -\frac{1}{\mathrm{i}\omega L}\left(1 + \sum_{q=1}^{4} e^{-\mathrm{i}k_q}\right) & \mathrm{i}\omega C + \frac{9}{\mathrm{i}\omega L} + \frac{\mathrm{i}}{\mathrm{i}\omega L} \end{pmatrix} \tag{S70}$$

$$= -\frac{1}{\mathrm{i}\omega L}\begin{pmatrix} -\left(9 + 4\mathrm{i} - \omega^2 LC\right) & 1 + \sum_{q=1}^{4} e^{-\mathrm{i}k_q} \\ 1 + \sum_{q=1}^{4} e^{\mathrm{i}k_q} & -\left(9 + 4\mathrm{i} - \omega^2 LC\right) \end{pmatrix}. \tag{S71}$$

Choosing $\omega^2 = 9/LC$ reduces the real part of the diagonal elements to zero in Eq. (S63). The remaining imaginary part on the diagonal only induces a constant shift of the spectrum and therefore does not alter the band structure under consideration in a qualitative sense.

SUPPLEMENTARY SECTION S VIII EXPERIMENTAL REALIZATION OF THE PHASE ELEMENT

A detailed circuit diagram is presented in Fig. S9, with the actual circuit board shown in Fig. S10. The phase element consists of four analog multipliers of type AD633 by Analog Devices Inc. The outputs W of the upper two multipliers are connected to two parallel inductors of type SRR7045-471M, with a nominal inductance of 470 μH at 1 kHz, forming inductance L. The outputs of the lower two multipliers are connected to a 50 Ω PTF6550R000BYBF resistor with a 50 Ω Bourns 3296W500 potentiometer in series. This combination of equally sized resistances and reactances allows for counter-rotating phase-variable impedances between V_1 and V_2, as desired. The Z inputs are used for manual DC offset compensation. To set the offset a high ohmic potentiometer is inserted between the positive and negative supply lines and its output range is down scaled by a voltage divider for precise adjustability. Therefore the divider consists of a 50 kΩ Bourns 3299W503 potentiometer ($R_{Z,2}$) between the supply voltages in combination with a 300 kΩ Yageo MFR-25FTF52-300K resistor ($R_{Z,3}$) and a 1 kΩ Bourns 3296W102 resistor ($R_{Z,1}$) to ground is used. The inputs for the supply voltages of the multipliers are buffered with 1 μF Murata GRM55DR72D105KW01L capacitors (C_b) to ground to not let high frequency signals, which the supply lines might have picked up, couple into the multipliers. The signals V_a and V_b are fed into the X inputs of the multipliers, whereas the signals at V_1 and V_2 are fed into the Y inputs, leading to the transfer function derived above. The DC-signals V_a and V_b where applied to the circuit by arbitrary waveform generators by Keysight of series type 33210A and 33500B. The connectors V_1 and V_2 are then coupled to connectors of one unit cell consisting of four parallel 47 nF Yageo CC0603MRX7R8BB473 capacitors to ground per site and one 470 μH SRR7045-471M inductor as coupling between the unit cell sites. The measurements took place at a frequency of 54.695 kHz with a signal of 1 Vpp and was recorded by three lock in amplifiers of type MFIA by Zurich Instruments, where two of the lock in amplifiers measured the voltages at the unit cell sites A and B respectively and the third lock in amplifier measured the current flowing into the circuit by taking a differential voltage measurement over a 12 Ω shunt resistor, consisting of a 10 Ω Yageo MFR200FRF52-10R resistor and a 2 Ω Yageo PNP5WVJT-73-1R resistor in series, connected to the signal output.

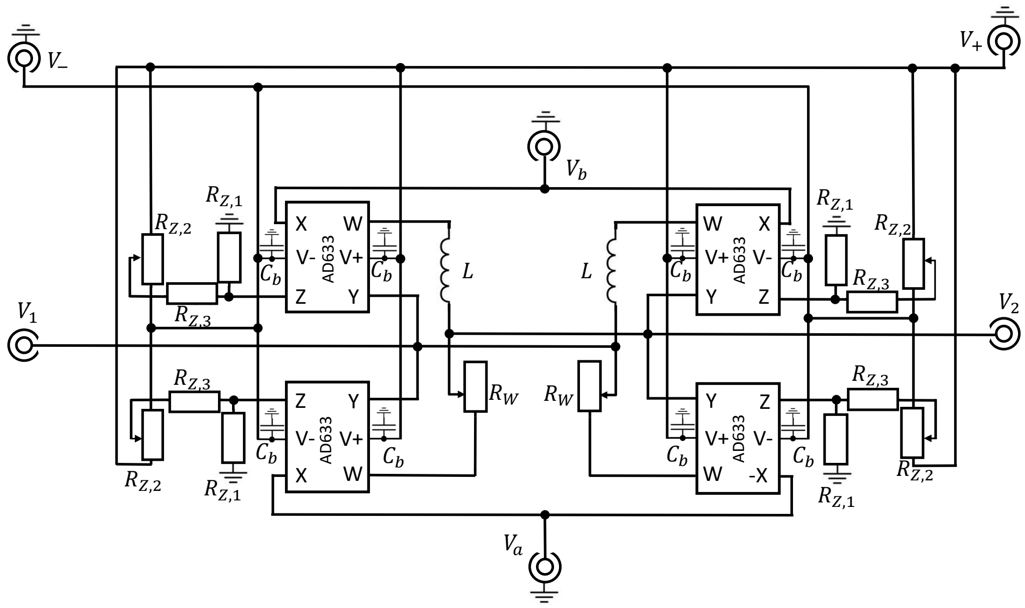

[h!]

FIG. S9. **Complex-phase element (detailed).** We show the detailed circuit diagram of the implementation of a boundary phase element using analog multipliers to enable continuous phase tuning. Four analog multipliers built the core of this element. The voltages V_a and V_b which cause the phase tuning are fed into the X inputs of the upper and lower multipliers respectively. The outputs W of the upper two multipliers are connected inductors, whereas the outputs of the lower two multipliers are each connected to a tunable resistor, allowing for tunable phases as described in the derivation of the element's Laplacian. The resistors $R_{Z,1}$, $R_{Z,3}$ and the potentiometer $R_{Z,2}$ are used for DC offset compensation. The supply voltage to the multipliers is connected via the connectors V_+ and V_-. The lines of the supply voltages are connected to capacitors to ground, to avoid high frequency signals to couple into the multipliers via these inputs. Via the connectors V_1 and V_2 the phase tuned signal is fed into an attached unit cell, which is not depicted.

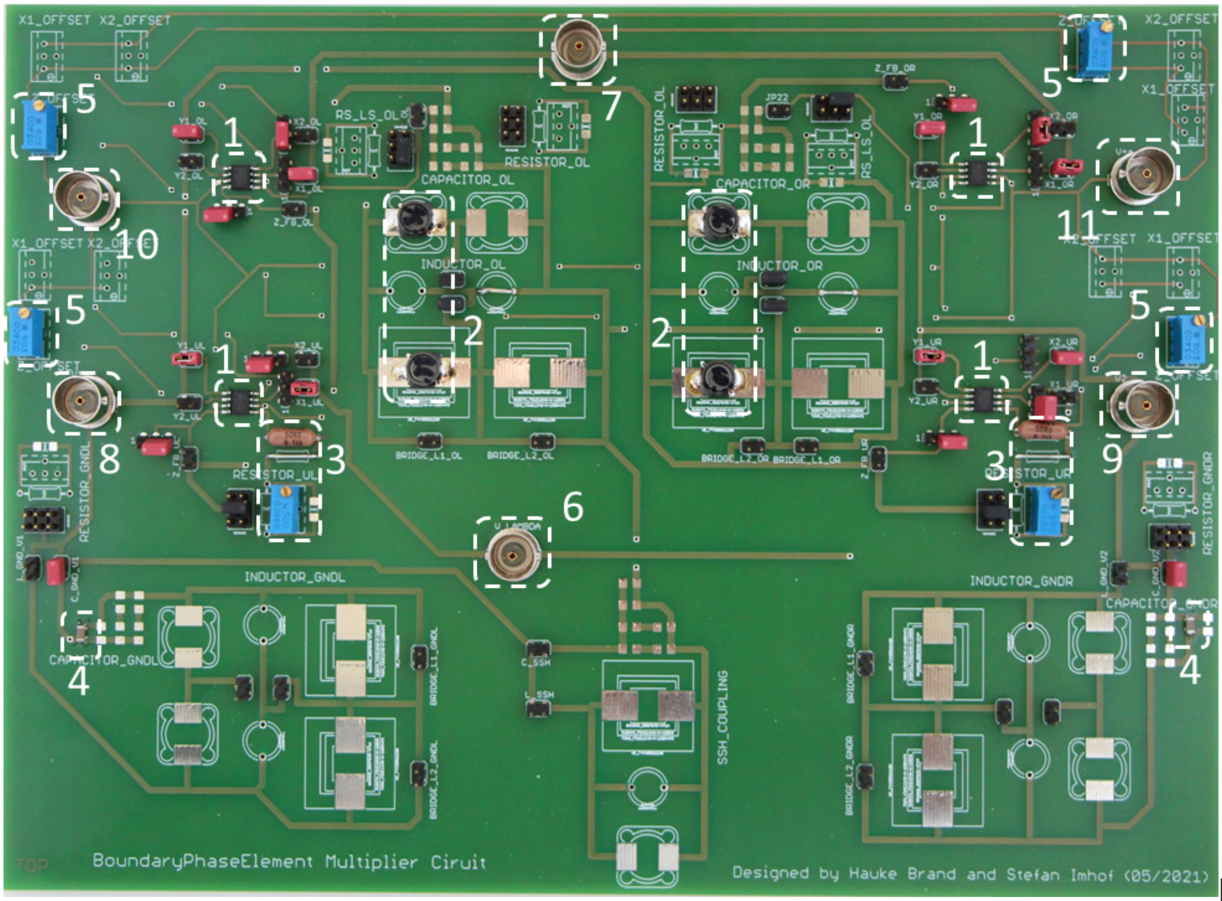

[h!]

FIG. S10. **Complex-phase element (circuit implementation).** 1) AD633 analog multipliers. 2) Two 470 μH inductors in of type SRR7045-471M in parallel. 3) 50 Ω PTF6550R000BYBF resistor with a 50 Ω Bourns 3296W500 potentiometer in series. 4) Capacitor of 47 nF of type Yageo CC0603MRX7R8BB473 to ground. 5) 50 kΩ Bourns 3299W503 potentiometer as part of a voltage divider connected to Z input of the respective multiplier (the other resistors of the divider are found on the back of the board). 6) BNC input for V_b. 7) BNC input for V_a. 8) & 9) BNC connectors to the nodes of the unit cell. 10) & 11) BNC input for negative and positive supply voltage respectively.

SUPPLEMENTARY REFERENCES

* iboettch@ualberta.ca

[1] I. Boettcher, A. V. Gorshkov, A. J. Kollár, J. Maciejko, S. Rayan, and R. Thomale, Crystallography of Hyperbolic Lattices, Phys. Rev. B **105**, 125118 (2022).

[2] J. Maciejko and S. Rayan, Hyperbolic band theory, Sci. Adv. **7** (2021).

[3] J. Maciejko and S. Rayan, Automorphic Bloch theorems for hyperbolic lattices, Proc. Natl. Acad. Sci. U.S.A. **119**, e2116869119 (2022).

[4] A. J. Kollár, M. Fitzpatrick, and A. A. Houck, Hyperbolic lattices in circuit quantum electrodynamics, Nature **571**, 45 (2019).

[5] C. Liu, W. Gao, B. Yang, and S. Zhang, Disorder-Induced Topological State Transition in Photonic Metamaterials, Phys. Rev. Lett. **119**, 183901 (2017).

[6] S. Yu, X. Piao, and N. Park, Topological Hyperbolic Lattices, Phys. Rev. Lett. **125**, 053901 (2020).

[7] P. Bienias, I. Boettcher, R. Belyansky, A. J. Kollar, and A. V. Gorshkov, Circuit Quantum Electrodynamics in Hyperbolic Space: From Photon Bound States to Frustrated Spin Models, Phys. Rev. Lett. **128**, 013601 (2022).

[8] M. Conder, Trivalent (cubic) symmetric graphs on up to 2048 vertices, `https://www.math.auckland.ac.nz/~conder/symmcubic2048list.txt` (2006), [Online; Accessed: 2022-02-07].

[9] A. M. Turner and A. Vishwanath, Beyond Band Insulators: Topology of Semimetals and Interacting Phases, in *Contemporary Concepts of Condensed Matter Science*, Vol. 6 (2013) pp. 293–324.

[10] M. S. Rudner and N. H. Lindner, The Floquet Engineer's Handbook, arXiv:2003.08252 (2020), arXiv:2003.08252.

[11] T. Oka and H. Aoki, Photovoltaic Hall effect in graphene, Phys. Rev. B **79**, 081406 (2009).

[12] T. Kitagawa, T. Oka, A. Brataas, L. Fu, and E. Demler, Transport properties of non-equilibrium systems under the application of light: Photo-induced quantum Hall insulators without Landau levels, Phys. Rev. B **84**, 235108 (2011).

[13] K. Gottfried and T.-M. Yan, *Quantum Mechanics: Fundamentals* (Springer, New York, 2003).
